# Deposition of Antimicrobial Copper-Rich Coatings on Polymers by Atmospheric Pressure Jet Plasmas

**DOI:** 10.3390/ma9040274

**Published:** 2016-04-07

**Authors:** Jana Kredl, Juergen F. Kolb, Uta Schnabel, Martin Polak, Klaus-Dieter Weltmann, Katja Fricke

**Affiliations:** Leibniz Institute for Plasma Science and Technology (INP Greifswald e.V.), Felix-Hausdorff-Str. 2, Greifswald 17489, Germany; jana.kredl@inp-greifswald.de (J.K.); juergen.kolb@inp-greifswald.de (J.F.K.); uta.schnabel@inp-greifswald.de (U.S.); polak@inp-greifswald.de (M.P.); weltmann@inp-greifswald.de (K.-D.W.)

**Keywords:** cold atmospheric pressure plasma, DC plasma jet, polymer, antimicrobial activity, cooper, *Staphylococcus aureus*, acrylonitrile butadiene styrene

## Abstract

Inanimate surfaces serve as a permanent reservoir for infectious microorganisms, which is a growing problem in areas in everyday life. Coating of surfaces with inorganic antimicrobials, such as copper, can contribute to reduce the adherence and growth of microorganisms. The use of a DC operated air plasma jet for the deposition of copper thin films on acrylonitrile butadiene styrene (ABS) substrates is reported. ABS is a widespread material used in consumer applications, including hospitals. The influence of gas flow rate and input current on thin film characteristics and its bactericidal effect have been studied. Results from X-ray photoelectron spectroscopy (XPS) and atomic force microscopy confirmed the presence of thin copper layers on plasma-exposed ABS and the formation of copper particles with a size in the range from 20 to 100 nm, respectively. The bactericidal properties of the copper-coated surfaces were tested against *Staphylococcus aureus*. A reduction in growth by 93% compared with the attachment of bacteria on untreated samples was observed for coverage of the surface with 7 at. % copper.

## 1. Introduction

The adherence of microorganisms on contact surfaces and its persistence for weeks or even months is a main cause of cross-contaminations and spread of infections in healthcare and food processing facilities [1,2,3,4]. The problem is emphasized by increasing the presence of microorganisms that have become resistant to a number of different antibiotics. The infection of surgical wounds by *Staphylococcus aureus* is a common problem in hospitals and its ability to develop resistance to antibiotics contributes to the danger to patients [5]. Despite hand-washing campaigns and routine cleaning, infection rates remain high. This has raised the need to combat pathogenic microorganisms to lower the risk of acquiring infections. A promising and effective strategy to prevent spreading of microorganisms in hygiene-sensitive areas is the refinement of inanimate surfaces with antimicrobial properties. Among different strategies, are contact-mediated killing metals like copper (Cu) and silver gaining in interest as self-sanitizing material. A further advantage in antimicrobial metals is that small concentrations are already sufficient to significantly inhibit the metabolism of bacteria, and thus achieve high anti-microbial efficacies (e.g., copper is recognized by the United States Environmental Protection Agency (EPA) as being able to continuously kill more than 99.9% of bacteria that cause hospital-acquired infections in 2 h [6]). Since copper-containing proteins are involved in cell metabolism, and, hence, copper in low concentration is an essential trace element in living organisms [7], there is little risk to patients and consumers. However, the redox properties of copper can cause some damage that has been studied for years [8,9]. The inactivation of microorganisms exposed to Cu has been attributed to the following mechanisms: generation of reactive oxygen species that causes oxidative stress, membrane damage due to the loss of structural integrity, and alteration to the conformational structure of proteins or degradation of the cellular DNA and/or RNA [10]. Materials with Cu doping are already used in medical instruments and sanitary facilities. Thus far, 500 copper alloys were registered as legally permitted antimicrobial materials. A variety of methods have been developed to deposit copper, as metal or as oxide, on surfaces, such as soaking, sol-gel coating, chemical washing, and atmospheric and vacuum coating. Plasma-based techniques are widely used, wherein the most frequently used processes for thin film deposition are based on low-pressure physical vapor deposition (PVD)—for instance, magnetron sputtering [11], plasma-immersion ion implantation [12] and chemical vapor deposition (CVD) using organometallic precursors [13]. Recent progress in the development of atmospheric pressure cold plasma technology has attracted increasing attention in thin film deposition. Intense research efforts are directed accordingly to identify new promising processes for the development of novel bioactive materials. Although there are some examples with respect to coatings with copper or silver nanoparticles, only a few reports can be found on the deposition of metal-containing thin films by using low temperature atmospheric pressure plasma. For instance, Beier *et al.* showed the antimicrobial effect of silver-containing silicon oxide layers generated by atmospheric pressure plasma chemical vapor deposition by introducing a silver nitrate solution into the gas discharge [14]. This aerosol-assisted process, in which an inorganic nanoparticle-containing dispersion is atomized and injected in atmospheric pressure plasma, is also reported by Fanelli *et al.* [15]. Another novel approach is the synthesis of copper particles by evaporating and sputtering bulk material from the powered electrode, which is also subject of the present work [16]. The advantage of the aforementioned plasma process is the delivery of metallic particles in high purity, which was also studied by Lazea-Stoyanova *et al.* by using an atmospheric radiofrequency plasma jet [17]. For this study, a non-thermal air operated DC plasma jet was used to deposit copper films on temperature labile acrylonitrile butadiene styrene (ABS) at room temperature [18]. ABS was selected for this study because of its increasing utilization as medical polymer. Due to their processability, recyclability, and mechanical properties, polymeric materials have become an integral part in the healthcare industry [19]. Moreover, today’s ongoing trend towards replacing conventional materials, such as metals, in medical devices by high performance polymers leads to many innovative materials. An essential contribution for medical polymers are styrenic polymers. Their physical and chemical properties meet the demands for many applications covering medical devices up to pharma packaging. Among these materials, ABS is the most important of the styrene copolymers [20]. The latest technical developments enable the production or grafting of ABS with high added value required for different applications. By incorporating fillers or nanoparticles, processing properties, and the mechanical resistance of ABS can be improved. For example, the thermal stability and tensile strength can be increased by adding silica nanoparticles [21], by copolymer composites of metal hydroxide nanorods and graphene nanosheets [22] or by the incorporation of carbon nanotubes and copper particles [23] whereas silver-containing powder have been added to achieve antibacterial properties [24,25]. However, plasma treated polymers have been characterized in terms of different operating parameters, including air flow rate and input current. X-ray photoelectron spectroscopy (XPS) and atomic force microscopy (AFM) have been used to investigate the chemical composition and the size, as well as the distribution of deposited particles. The bactericidal properties of Cu-modified samples were evaluated against *Staphylococcus aureus (S. aureus*).

## 2. Results and Discussion

### 2.1. Chemical Characteristics

**Figure 1:**
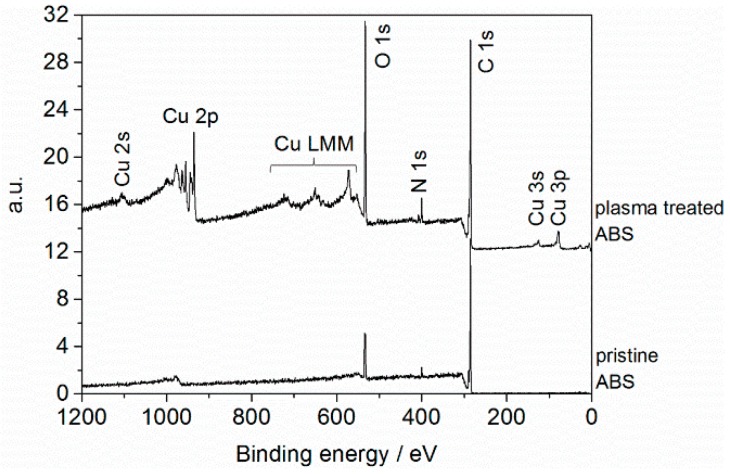
Survey spectra of pristine acrylonitrile butadiene styrene (ABS) and of ABS exposed to air plasma (operating parameters: *Q*_air_ = 8 slm, *I* = 30 mA, *t* = 432 s).

Plasma-induced surface modification of ABS was investigated by XPS analysis. Representative survey spectra of pristine ABS and ABS exposed to air plasma are shown in Figure 1.

**Figure 2:**
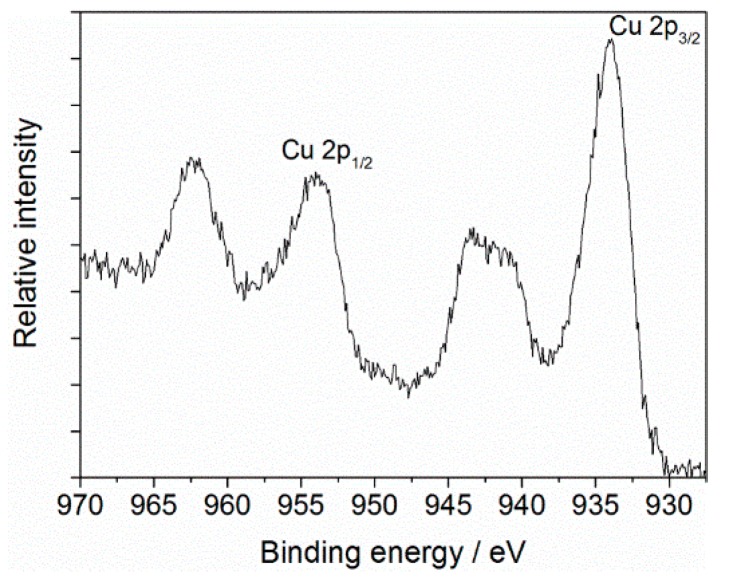
Cu 2p core level XP spectrum of ABS exposed to air plasma (operating parameters: *Q*_air_ = 8 slm, *I* = 30 mA, *t* = 432 s).

Figure 1 shows that the surface of pristine ABS is composed of carbon (C 1s at 285 eV), nitrogen (N 1s at 399 eV), and oxygen (O 1s at 532 eV). According to its chemical structure, ABS is an oxygen-free polymer, but the presence of surface-near oxygen has been reported frequently and may be related to additives used for the fabrication of this copolymer and to contaminations from ambient air (e.g., adsorbed water) [26,27,28]. However, XPS results reveal changes in the survey spectrum of ABS upon exposure to air plasma. New emission lines have been identified that are attributed to copper. The nature of the Cu in the films can be further analyzed from the Cu 2p region of the X-ray photoelectron (XP) spectrum. The corresponding core level Cu 2p spectrum (representative for all of the surfaces studied) in the 930–970 eV region is shown in Figure 2.

Intense Cu 2p_3/2_ and Cu 2p_1/2_ peaks are centered at 934 and 954 eV, respectively, along with shake-up features at ~942 and ~962 eV for Cu 2p_3/2_ and 2p_1/2_ core levels, the latter being indicative of Cu(II) state [29,30]. The peak positions and the relative intensities of the satellites from these levels are strongly related to the presence of copper oxides, in particular the formation of CuO and/or Cu(OH)_2_ phase at the surface is suggested [31,32].

**Figure 3:**
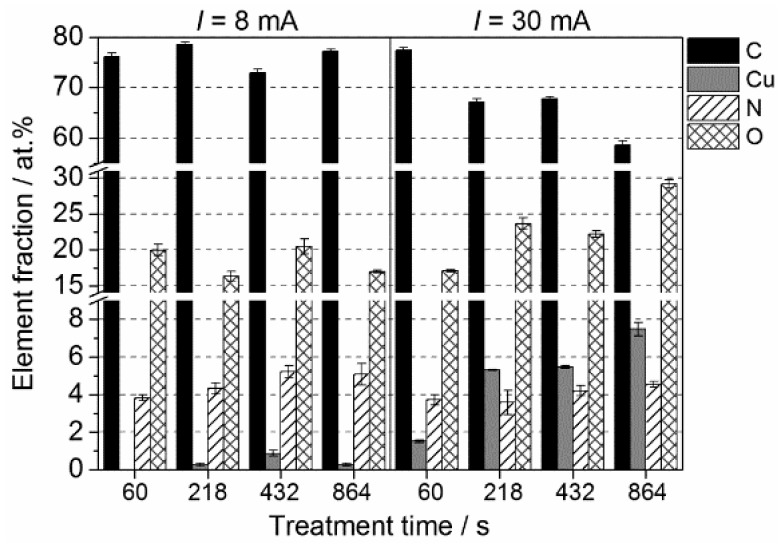
X-ray photoelectron spectroscopy (XPS) results: Atomic concentration (at. %) of carbon (C); copper (Cu); nitrogen (N); and oxygen (O) depending on treatment time and applied input current. Results are presented as averaged value from three measurements for each specimen. (Operating parameter: *Q*_air_ = 4 slm).

In order to understand the influence of experimental parameters on Cu thin film deposition, a thorough XPS analysis was conducted. Figure 3 shows the time-dependent variation of the elemental composition of plasma treated ABS specimens for input currents of 8 and 30 mA, respectively. It can readily be seen that the deposition of copper is poor at short treatment times. In detail, when using a low input current of 8 mA, Cu was detected only at treatment times above 218 s. Nevertheless, Cu is only present in trace amounts with a maximum of 0.8 at. %. Substantial changes in Cu content can be seen for input currents of 30 mA. Already after 60 s, a Cu amount of 1.5 at. % was detected. It is also evident that increasing the treatment time leads to an increased deposition of copper with a maximum of almost 8 at. % after 864 s. The Cu content, however, slightly decreased at low input currents after 864 s. In the operation of the plasma jet, different modes can be observed that can be characterized primarily as a self-pulsing operation and a true stable DC operation. The plasma characteristic is strongly dependent on the operating current, which, in turn, primarily determines the deposition of copper. For lower currents, a self-pulsing mode is likely the dominant process, while for higher currents, a DC operation is becoming more prevalent [33,34]. With the different discharge modes, plasma parameters are changing and affecting deposition rates consequently. According to these findings, data presented hereinafter were obtained for treatment times of 432 s while changing different process parameters.

**Figure 4:**
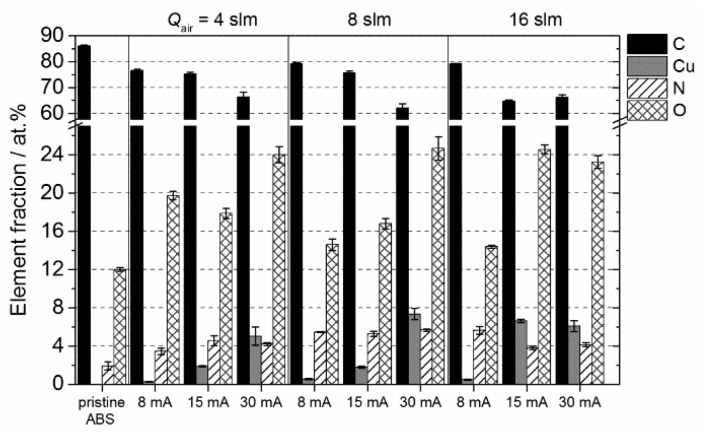
XPS results: Atomic concentration (at. %) of carbon (C); copper (Cu); nitrogen (N); and oxygen (O) depending on applied input current and gas flow rate. Results are presented as averaged value from three measurements for each specimen.

A comparison of the elemental composition of plasma treated ABS specimens at various input currents and air flow rates analyzed by XPS is depicted in Figure 4. After plasma treatment, the relative surface composition was altered significantly. Compared with pristine ABS, a notable reduction in carbon content is accompanied by a remarkable increase of copper and oxygen concentrations for all tested surfaces. Furthermore, an increase of the nitrogen concentration from initially 2 at. % up to 5.7 at. % was measured. Among the parameters investigated, the input current is the most influential on the elemental surface composition. The relative surface concentration of Cu was found to increase with higher operating current from 0.4 at. % for 8 mA up to 7.5 at. % Cu at 30 mA. A possible explanation for the present result is the energy provided to the plasma changes with increasing input current. Previous characterizations of the plasma reveal that the gas production rate was enhanced with rising input currents [34]. Consequently, since Cu species were provided into the plasma by sputtering of the electrode, it is most likely that, for high input currents, electron collision processes are becoming important, which influence the generation of reactive plasma species that favor the deposition of copper. In terms of the air flow rate, the results show that this process parameter only marginally effects the elemental composition when operating at 30 mA.

**Figure 5:**
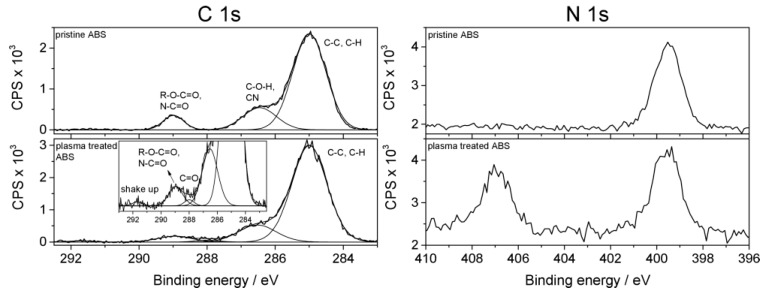
XPS high resolution C 1s spectra and N 1s core level spectra of pristine ABS and of ABS exposed to air plasma (operating parameters: *Q*_air_ = 8 slm, *I* = 30 mA, *t* = 432 s).

The chemical identification of functional groups has been conducted by high resolution XPS analysis. Figure 4 shows the curve-fitted spectra in the C 1s and N 1s regions for the surface of pristine and plasma treated ABS, respectively. In the case of C 1s spectra, it is apparent that only slight changes in the peak shape were induced. The C 1s spectra have been fitted with components assigned to functional groups according to the literature [26,35]. The C 1s envelope was fitted with three components: the peak at 285.0 eV is attributed to C–C and C–H bonds, the second component at 286.5 eV can be associated with either C–O–H or C≡N nitrile groups, and the peak at 289.0 eV is assigned to R–O–C=O carboxylic groups or to imide groups. After treatment, a new peak appears at 287.9 eV, which suggests the formation of C=O carbonyl groups and/or N–C=O amide groups. Hence, it was found that, depending on the process parameters, oxygen and nitrogen added by the air plasma forms new double bonds with carbon. In contrast, the N 1s spectrum was found to change after plasma treatment. In the case of pristine ABS, the N 1s core level spectrum shows a single peak at 399.7 eV, which is consistent with the binding energy value reported for C≡N functionalities present in the polymer backbone. As can be seen in Figure 5, a new peak appears at 407.0 eV after plasma treatment that may arise from oxidized species of nitrogen (e.g., nitro or nitrate groups) [36]. The formation of these new surface functionalities results from the reaction of surface radicals with nitric oxide, which is generated in the gas discharge [34].

Overall, functional groups remain on the surface after plasma treatment, but, additionally, the process slightly alters the surface chemistry due to the modification of the polymer chains and the removal of adsorbed contaminants. Along with surface chemical modification, changes in the wettability have been observed depending on the process parameters. ABS is characterized by a poor wettability with an averaged water contact angle of 81°. After plasma treatment, static water contact angle measurement revealed contact angles ranging from 62° at an input current of 15 mA and an air flow rate of 8 slm up to 29° at 30 mA and 16 slm. As described in the literature, the adhesion of a thin metallic layer depends on the surface chemistry and can be improved by surface functionalization [37,38,39]. Therefore, an increase in surface functionality and in wettability may contribute to the deposition of copper-containing films.

### 2.2. Topographical Characteristics

**Figure 6:**
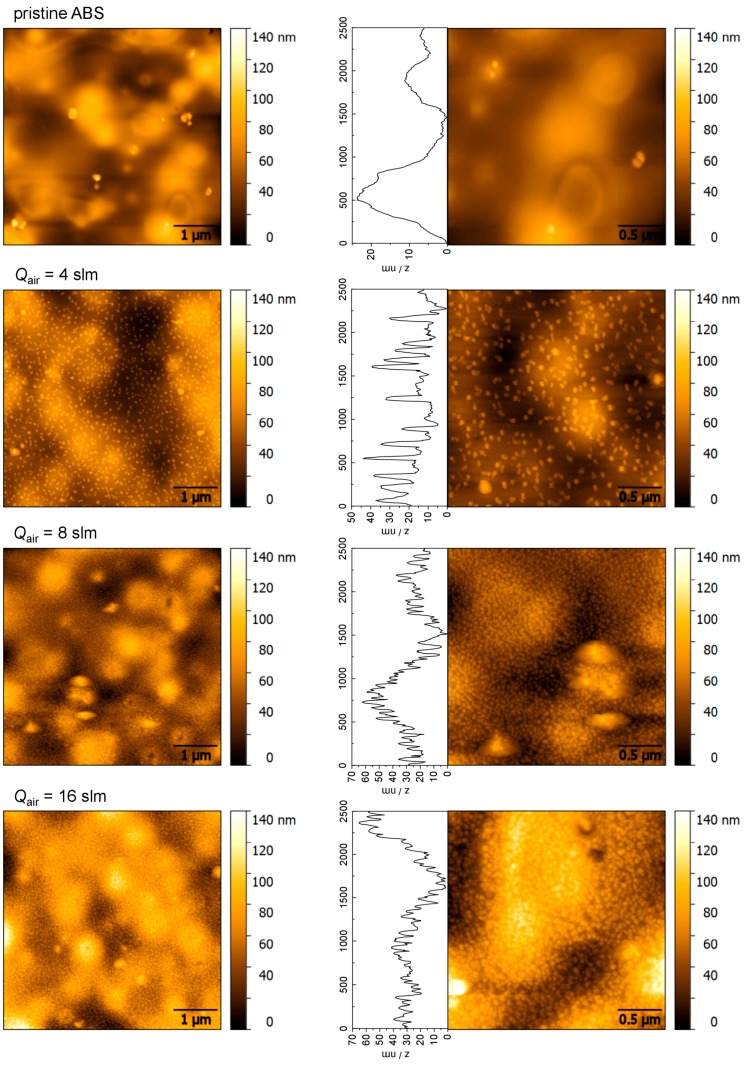
Atomic force microscopy (AFM) images (5 µm × 5 µm and 2.5 µm × 2.5 µm) and height profiles of pristine ABS and of ABS after plasma treatment for different air flow rates (operating parameters: *I* = 30 mA, *t* = 432 s).

The bactericidal effect of metal nanoparticles has been attributed to their small size and high surface to volume ratio, which allows them to interact closely with microbial membranes [40]. The surface topography as well as the distribution of deposited copper particles of plasma treated surfaces was examined by using AFM. To get a better understanding of CuO thin film growth, AFM analysis of surfaces treated with various air flow rates was conducted according to the different amount of deposited Cu. Representative micrographs of pristine ABS and of ABS exposed to different air flow rates at a given input current of 30 mA are displayed in Figure 6. Additionally, in order to evaluate the size of deposited particles, height profiles have been extracted from a scanning area of 2.5 µm × 2.5 µm. However, the topography of pristine ABS is characterized by a well-defined hill-and-valley structure with hills of several µm in diameter, which can be seen in the corresponding height profile. There are also a few dust particles observed on the surface. After plasma treatment, a more irregular surface topography is evident, which is characterized by the presence of copper nanoparticles with a size of several tens of nanometer. Clearly, the variation of the flow rate affects the copper surface coverage: For surfaces of 4 slm, plasma treatment, nanosheet-shaped particles of different diameters have been identified. It has further been found that particles are deposited in islets with no specific distribution pattern. The approximate particle size was estimated to be in the range from 40 to 100 nm with distances in the order of 100−500 nm, which is much smaller than the size of pathogenic bacteria. For instance, the size of bacterial species ranges from 0.2 µm to the largest cells of sulfur bacteria with a diameter of 750 µm, whereas for Staphylococci, diameters of 0.5−1.5 μm are reported [41,42]. However, by applying higher flow rates, the number of deposited particles was increased, which led to a particle distribution in a close-packed arrangement. These findings are even more pronounced at the highest flow rate of 16 slm. The corresponding height profiles for surfaces of 8 and 16 slm reveal that the particles have a size ranging from 10 to 80 nm in diameter. Furthermore, a coalescence of spherical surface features can be seen which initiates the forming of bigger compact particles, a process known as agglomeration.

**Figure 7:**
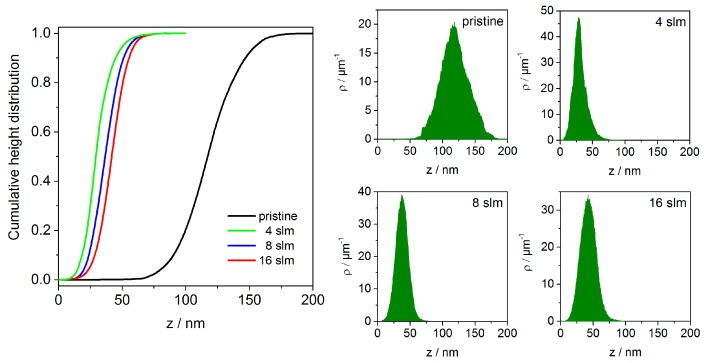
Cumulative height distribution plots (**left**) of the topographical images shown in Figure 6. The plots are normalized to show probability values from 0 to 1; (**right**) Corresponding histograms of pristine ABS and of ABS after plasma treatment obtained at different air flow rates (operating parameters: *I* = 30 mA, *t* = 432 s).

In order to estimate the particle size more precisely, histograms (statistical distribution of z-values) of AFM images presented in Figure 6 are investigated. For ease of comparison, the measured height distributions are also plotted as cumulative distribution, which is the integral of the density. As can be seen in Figure 7, the average height of non-treated ABS was approximately 118 nm with values ranging between 60 and 180 nm. These values correspond to the hilly landscape of the ABS substrate as well as to the presence of dust particles that originate from sample handling in ambient air. Therefore, processed data of plasma treated substrates presented in Figure 7 are obtained from flattened images. Hence, comparing the histograms of plasma treated surfaces depending on the air flow rates, a maximum height of 28, 37, and 42 nm for 4, 8, and 16 slm, respectively, was determined. The slight but significant change to higher z values with increasing flow rate indicates some agglomeration of round-shaped copper nanoparticles.

### 2.3. Antimicrobial Results

Based on the XPS analysis, the antimicrobial activity of copper-modified surfaces and the impact of different amounts of copper towards the Gram-positive bacteria *Staphylococcus aureus* after two hours of incubation were evaluated. In particular, the percentage reduction of colony forming units for each set of samples was calculated to express the change of the microbial population relative to the pristine ABS. The change was determined as follows:

(1) $$\%\text{ Reduction}=\;\frac{\left(N_{0}-N\right)\times 100}{N_{0}}.$$

*N*_0_ is the number of colony forming units on pristine ABS, and *N* is the number of colony forming units on plasma treated samples.

**Figure 8:**
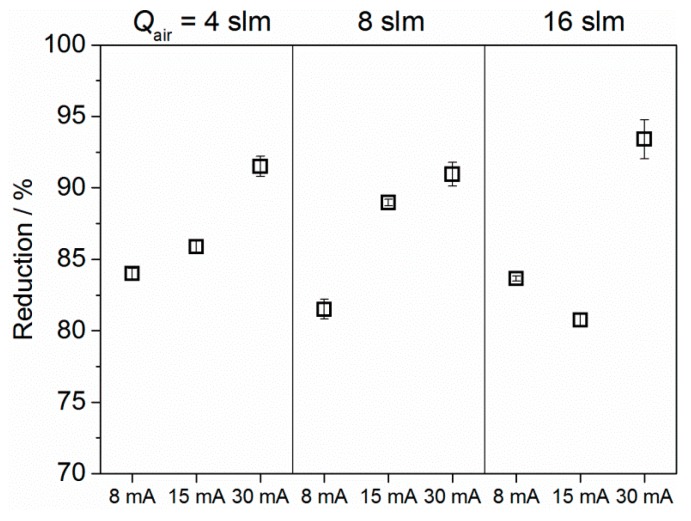
Effect of air flow rate and input current on the inactivation of *Staphylococcus aureus*, which is presented in percentage reduction. Values are the average of triplicate. The number of colony forming units on pristine ABS was *N*_0_ = 1 × 10^7^, and the detection limit was 99.9999%.

It can be readily seen from the results depicted in Figure 8 that the deposited copper oxide films demonstrate biocidal activity. In detail, for a low input current of 8 mA, a reduction of up to 84% was achieved among the different air flow rates investigated. At medium input current of 15 mA, an increase of the reduction with a maximum of 87% can be observed. By increasing the input current to 30 mA, the highest percentage reduction of about 93% was obtained in this study. According to the XPS results presented in Figure 4, the following assumption can be made: the inactivation of *S. aureus* after contact with copper-coated ABS surfaces was significantly affected by the concentration of copper. Greater antimicrobial activity was noticed for surfaces exposed to an input current of 30 mA, which led to a surface coverage of Cu concentrations between 5 and 7 at. %. Regarding the inactivation mechanisms, it should be noted that copper is a contact-killing agent by interacting either directly with the cellular membrane or intracellularly. In this way, the mechanism for the antimicrobial behavior of the copper-coated ABS might be directly related to the release rate of copper. The release behavior of copper in turn, is influenced, for instance, by the layer thickness, size of copper particles, and the aqueous environment [43]. Regarding the thickness of the copper oxide films, it can be estimated that the deposits obtained at input currents of 30 mA are the size of a few nanometers only. Moreover, for the lowest flow rate of 4 slm, AFM images (Figure 6) revealed a non-uniform film, which was characterized by single copper particles. Apparently, due to the small amount of copper deposited at an operating current of 8 mA, it is most likely that no copper is left on the surface after two hours, which results in low inactivation rates. Hence, in this study, a maximum reduction of 93% has been achieved for plasma treated surfaces with the highest copper concentration. These results suggest further studies to optimize deposited copper concentrations with respect to an improved bactericidal activity. In addition, further research is required to understand the correlation between antibacterial effect and copper release rate.

## 3. Materials and Methods

### 3.1. Polymeric Material

The experiments were carried out on ABS plates of commercial grade with a size of 10 mm × 10 mm and a thickness of 1.5 mm, which were purchased from Goodfellow GmbH (Bad Nauheim, Germany).

### 3.2. Plasma Source and Plasma Treatment

**Figure 9:**
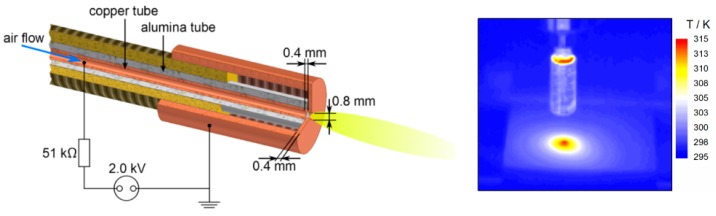
(**Left**) Schematic design of the micro hollow discharge geometry of the DC plasma jet; (**Right**) Surface temperature of ABS during plasma treatment.

The design of the plasma jet (shown in Figure 9) is based on a micro hollow discharge geometry that was presented in earlier studies [16,44,45]. The jet consists of an inner electrode made from a copper tube with an inner diameter of 0.8 mm and an outer diameter of 1.6 mm. This cathode is isolated from the outer grounded electrode by an alumina ceramic tube with an inner diameter of 1.6 mm and a wall thickness of 0.8 mm. The ground electrode enclosing the ceramic is sealed by screw cap. In the center of the cover is a small hole with a diameter of 0.8 mm through the 0.4 mm thick lid. The ceramic tube is 0.4 mm longer than the inner high voltage electrode, which keeps it separated from the cover.

**Figure 10:**
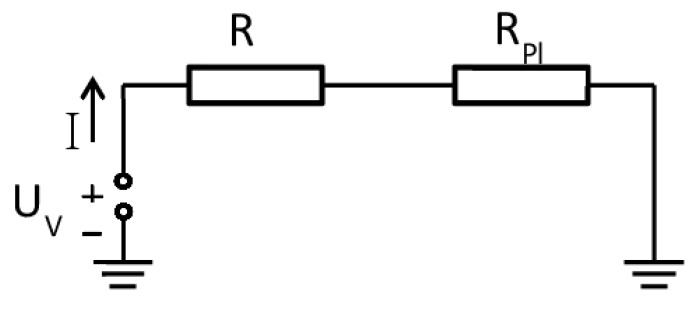
Equivalent circuit diagram of the DC plasma jet.

Plasma is generated in the gap between the inner and outer electrode when a negative voltage of 2 ± 0.2 kV from a DC high voltage power supply (PS FX06R50, Glassman High Voltage Inc., High Bridge, NJ, USA) is applied. A resistor of 50 kΩ connected in series limits the current (see Figure 10). For the experiments, the current was adjusted to 30 mA. Voltage across the 50 kΩ resistor was monitored with a high voltage probe (P5100A, Tektronix, Beaverton, OR, USA). From current and voltage drop across the resistor, the power dissipated in the plasma is determined to be about 15 W. Compressed air was used as operating gas flowing through the inner copper tube electrode. The gas flow rate was controlled by a mass flow controller (MF1C01324CMAV0, MKS Instruments, Andover, MA, USA) and a four-channel mass flow and pressure display unit (647C-4-R-1-N, MKS Instruments). With a gas flow of 8 slm, the plasma is expelled from the 0.8 mm orifice in the cap generating an afterglow plasma plume of 5–10 mm. The temperature of the plasma between the electrodes is about 1500 K, which is the approximated value for the gas temperature in a micro hollow cathode discharge in air at atmospheric pressure [46]. However, due to the air flow, the plasma plume temperature at 10 mm distance in axial direction from the orifice is already close to room temperature. This gas temperature of the expelled plume was measured with an optical interference thermos-sensor (UMI 4, FISO Technologies, Quebec, QC, Canada). For the experiments, the plasma jet device was fixed along the *z*-axis of a stepper motor controlled xyz-stage (FF500/CNC, Proxxon GmbH, Föhren, Germany) at a distance of 10 mm to the ABS samples. For a uniform exposure, the jet was moving in a meandering way, covering an area of 20 mm × 20 mm (with the sample sitting in the middle) across the sample twice. The second meandering route was perpendicular to the first. To vary treatment times, the stepper-motor speed and the distance between the passes of the meander can be changed.

### 3.3. Surface Analysis

The chemical surface composition was determined by high-resolution scanning XPS. Spectra were acquired using an Axis Ultra DLD electron spectrometer (Kratos Analytical, Manchester, UK) with a monochromatic Al Kα source (1486 eV). Charge neutralization was implemented by low energy electron injected into the magnetic field of the lens from a filament located directly atop the sample. The instrument was set to the medium magnification (field of view 2) lens mode and by selecting the slot mode, providing an analysis area of approximately 250 µm in diameter. Survey spectra and core level spectra of all identified elements were collected at a pass energy of 80 eV and for the highly resolved measured carbon (C 1s) peak the pass energy was set to 10 eV. Three different spots on each specimen were analyzed twice and averaged. Data acquisition and processing were carried out using CasaXPS software, version 2.14dev29 (Casa Software Ltd., Teignmouth, UK). Due to sample charging, the binding energy scale was corrected for all samples by setting the C 1s binding energy to 285.0 eV for the C–(H, C) component in the C 1s spectra. Concentrations are given in atomic percent (at. %).

AFM was performed with a scanning probe microscope diCP-II (Veeco Instruments, Santa Barbara, CA, USA) in the non-contact mode, especially tapping mode. An area of 5 µm × 5 µm and 2.5 µm × 2.5 µm was scanned using a pyramidal silicon tip doped with n-type phosphorus with a resonance frequency of 273–389 kHz and a force constant of 20–80 N/m (Veeco, RTESPA-CP, Mannheim, Germany). AFM-images were recorded on selected samples and processed using the open-source software Gwyddion 2.39 [47].

### 3.4. Antimicrobial Test

For microbiological experiments, *Staphylococcus aureus* DSM 799 (ATCC 6538) was used in concentrations of 10^7^ cfu/mL in sodium chloride solution (0.85%). *S. aureus* was obtained from the Leibniz Institute DSMZ-German Collection of Microorganisms and Cell Cultures (Braunschweig, Germany). The investigated specimen was UT RowePaedM SA 200 mm bulk/unsterile (Company RoweMed, Parchim, Germany) from ABS covered with copper as described above. The specimen was inoculated with 50 µL of the *S. aureus* suspension by pipetting. Afterwards, a drying step of 2 h under aseptic conditions followed. The recovery was done with nutrient solution (tryptic soy, Merck, Darmstadt, Germany). The specimens were given in 10 mL nutrient broth and shaken for 15 min at 350 rpm at room temperature. The recovery of survivors was realized using the surface-spread-plate count method with tryptic soy agar plates, and it was completed with an overnight cultivation in an incubator at 37 °C. The surface-spread-plate count method is a surface counting method employed for aerobic bacteria. For the test, 100 µL of all serial dilutions of the broth were plated out on the whole surface-area of the petri dish. Serial dilutions were performed as a 1 in 10 dilution. The detection limit of this procedure was 10 cfu/mL. If the number of microorganisms fell below the detection limit, *i.e.*, no viable microorganisms have been found, these values in the graphs were set at the detection limit.

## 4. Conclusions

The objective of this work was to evaluate the bactericidal properties of polymer surfaces modified by copper deposits from a cold atmospheric pressure plasma. XPS analysis revealed the surface functionalization of ABS along with the deposition of copper oxide films. It was shown that the deposited Cu concentration strongly depends on the input current (*i.e.*, energy dissipated in the plasma) and on the air flow rate. AFM images confirmed the deposition of copper particles at the nanoscale size. The described films showed their potential use as biocidal coating, which might be of great importance for the reduction of microbial load on inanimate surfaces to prevent, for instance, the transfer of infections in health care facilities and to protect materials from microbial degradation. Furthermore, since this is an environmentally friendly process for copper thin film deposition combined with low temperatures, it is compatible with various materials, including textiles and weaving, which provides advanced properties for tailored applications. Further studies will be focused on the deposition of copper-rich films on different materials.

## Abbreviations

The following abbreviations are used in this manuscript:

ABS	acrylonitrile butadiene styrene
DC	direct current
XPS	X-ray photoelectron spectroscopy
AFM	atomic force microscopy
EPA	Environmental Protection Agency
PVD	physical vapor deposition
CVD	chemical vapor deposition
slm	standard liter per minute
*S. aureus*	*Staphylococcus aureus*
